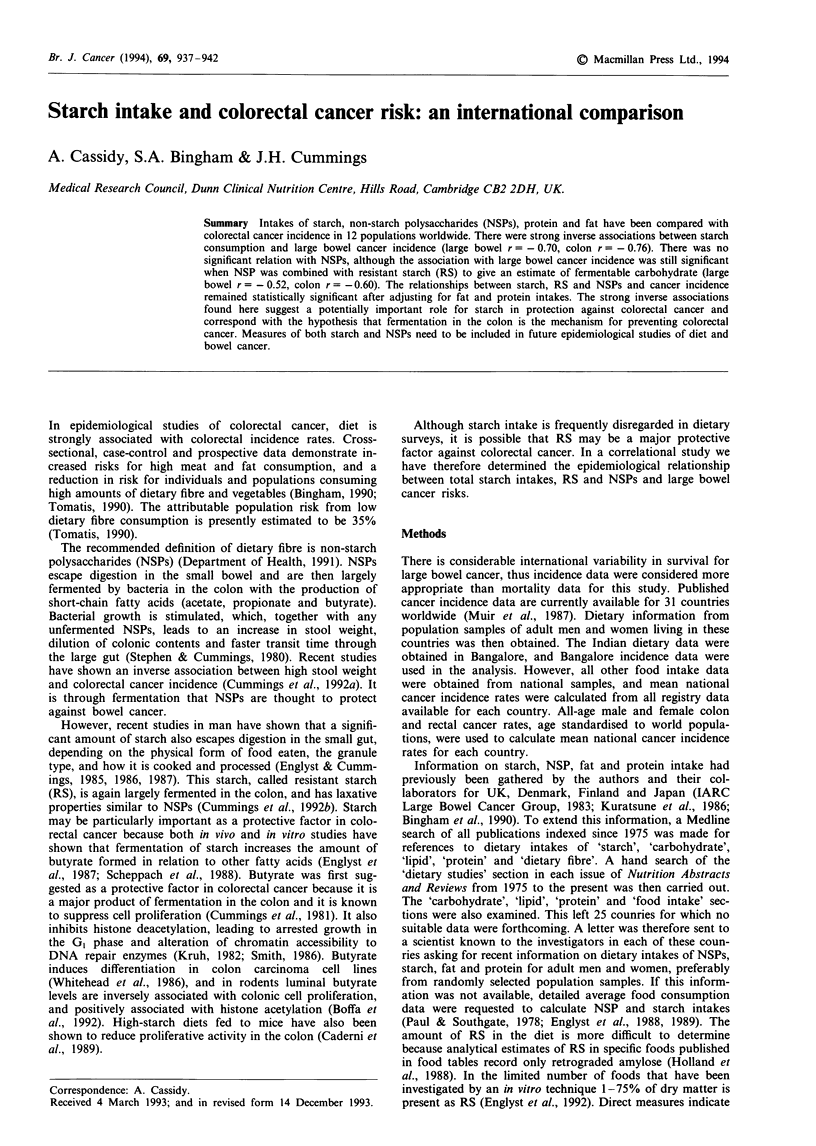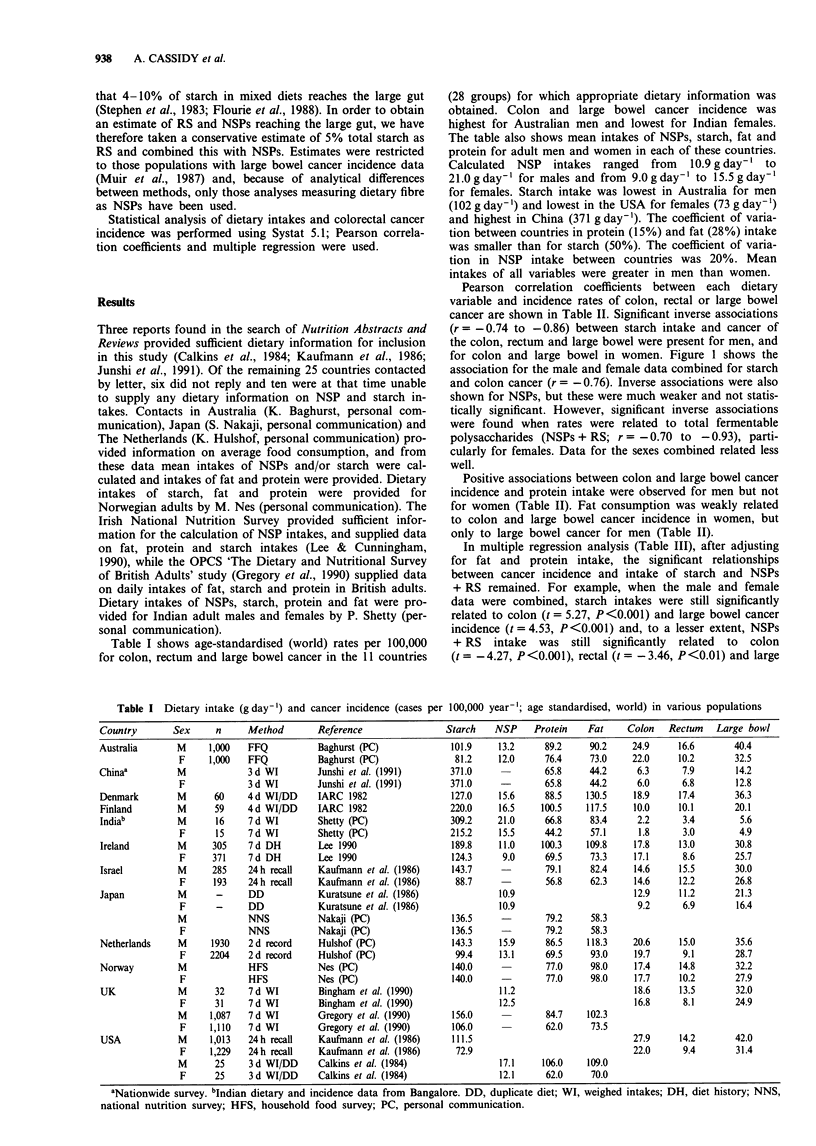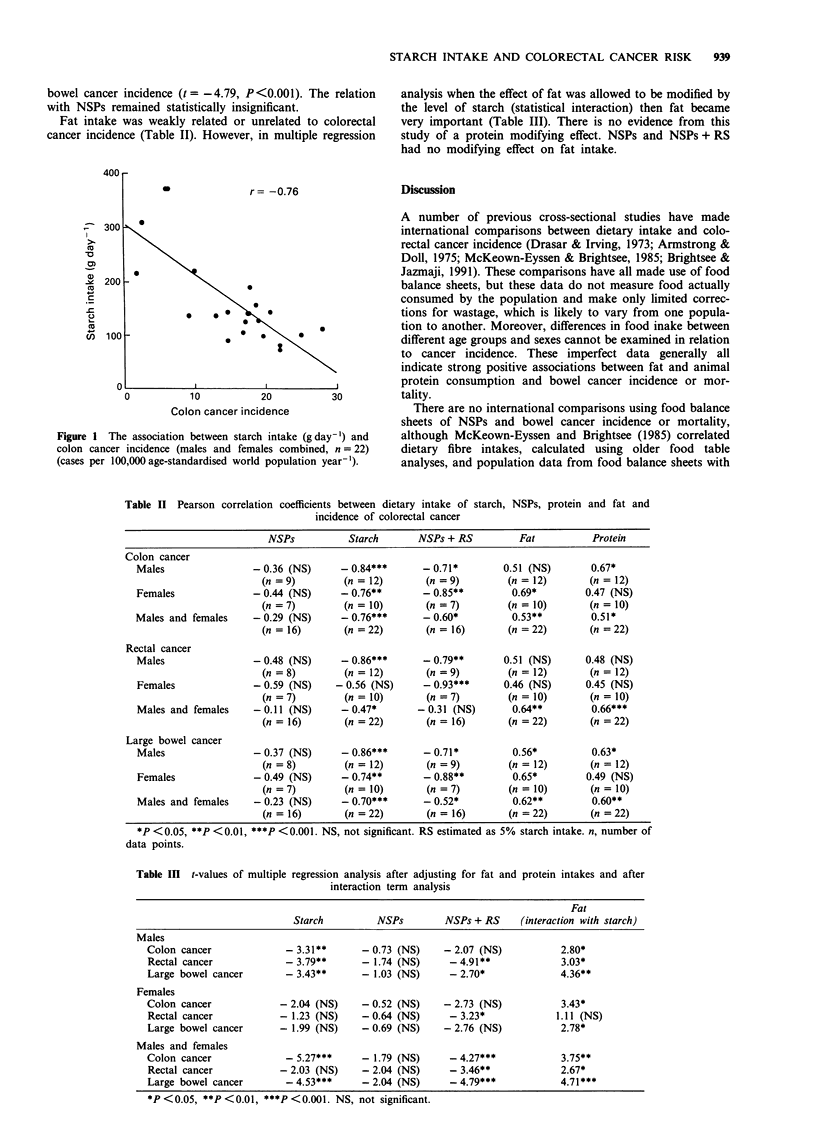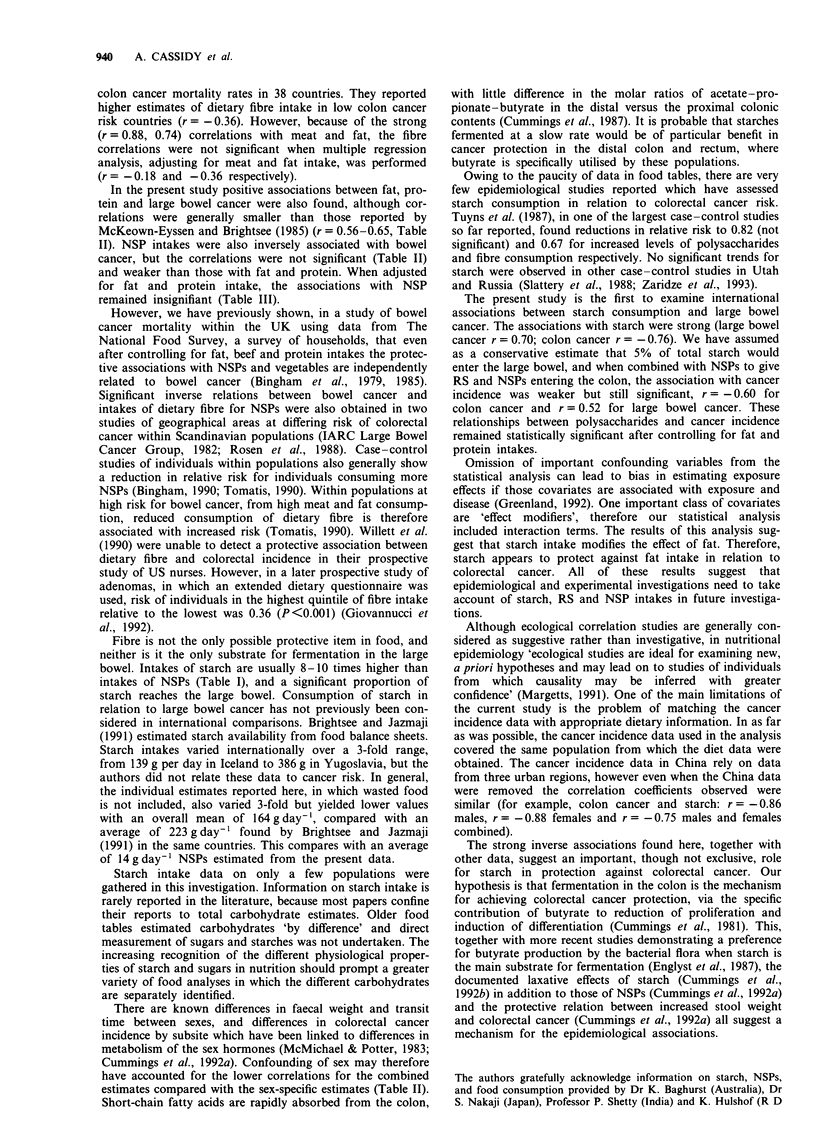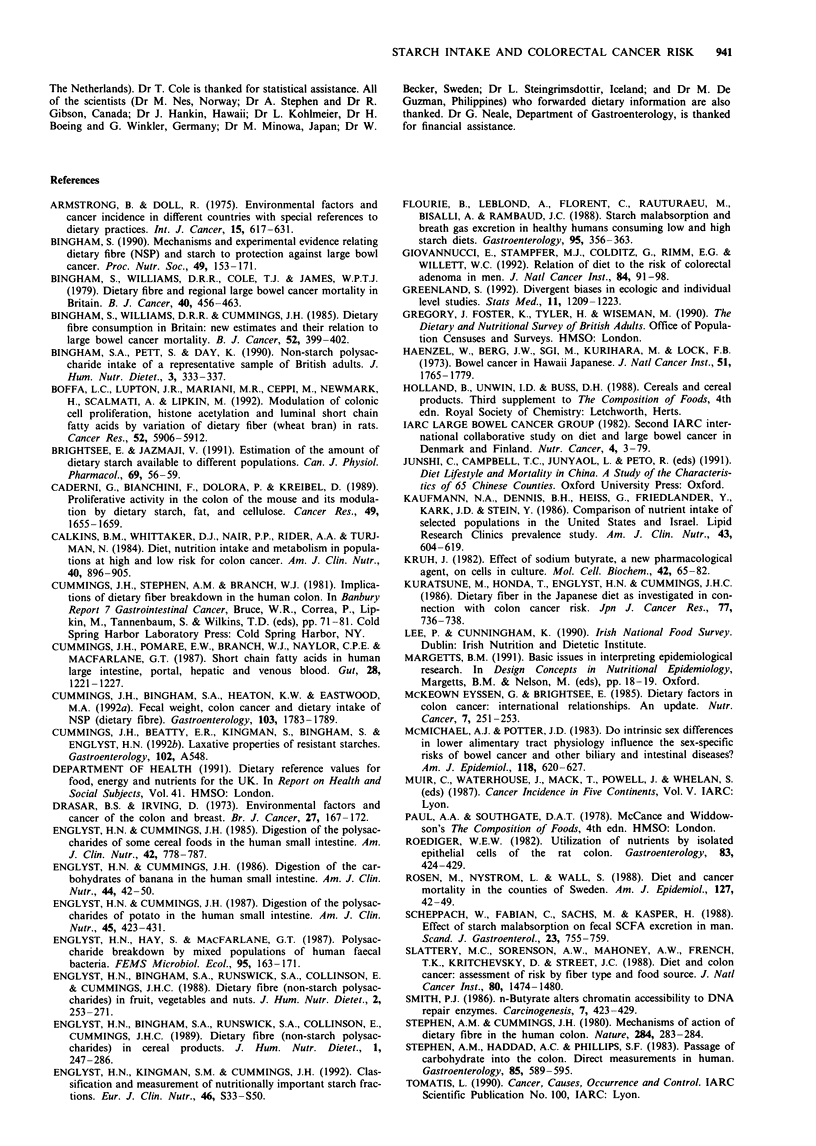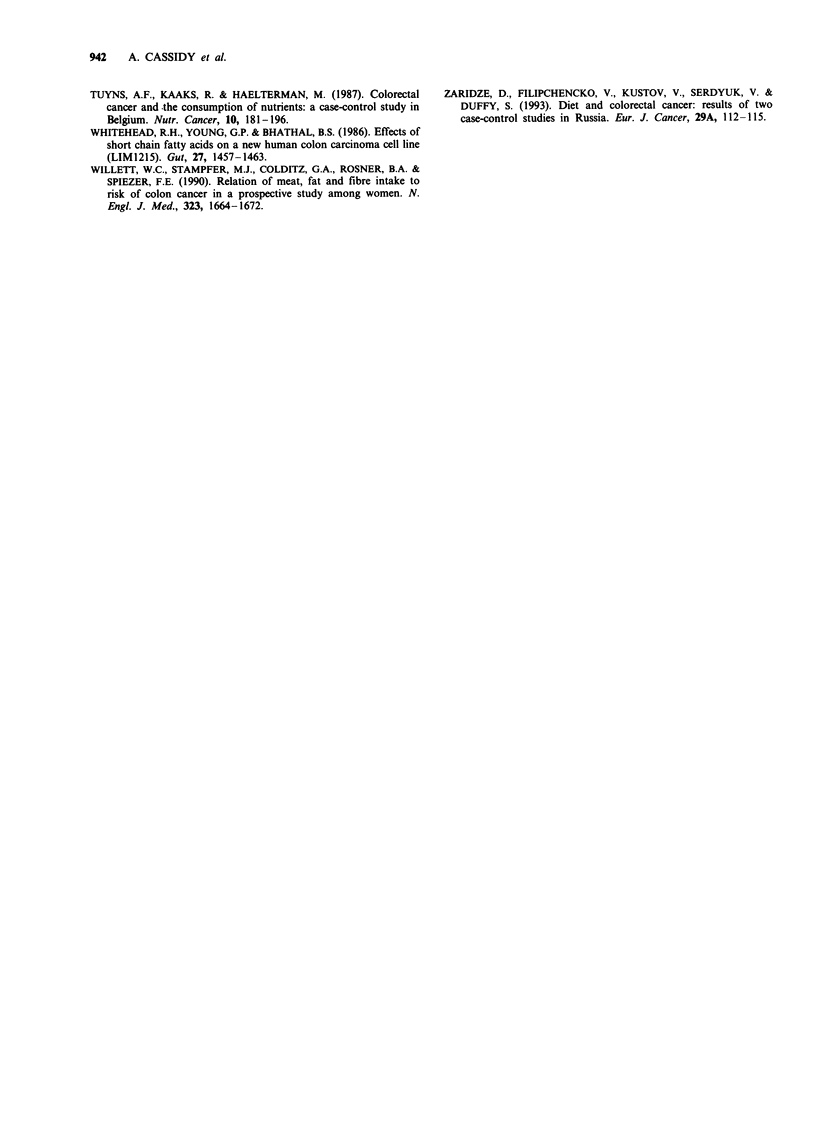# Starch intake and colorectal cancer risk: an international comparison.

**DOI:** 10.1038/bjc.1994.181

**Published:** 1994-05

**Authors:** A. Cassidy, S. A. Bingham, J. H. Cummings

**Affiliations:** Medical Research Council, Dunn Clinical Nutrition Centre, Cambridge, UK.

## Abstract

Intakes of starch, non-starch polysaccharides (NSPs), protein and fat have been compared with colorectal cancer incidence in 12 populations worldwide. There were strong inverse associations between starch consumption and large bowel cancer incidence (large bowel r = -0.70, colon r = -0.76). There was no significant relation with NSPs, although the association with large bowel cancer incidence was still significant when NSP was combined with resistant starch (RS) to give an estimate of fermentable carbohydrate (large bowel r = -0.52, colon r = -0.60). The relationships between starch, RS and NSPs and cancer incidence remained statistically significant after adjusting for fat and protein intakes. The strong inverse associations found here suggest a potentially important role for starch in protection against colorectal cancer and correspond with the hypothesis that fermentation in the colon is the mechanism for preventing colorectal cancer. Measures of both starch and NSPs need to be included in future epidemiological studies of diet and bowel cancer.


Br. J. Cancer (1994), 69, 937-942 © Macmillan Press Ltd., 1994

### Starch intake and colorectal cancer risk: an international comparison

A. Cassidy, S.A. Bingham & J.H. Cummings

*Medical Research Council, Dunn Clinical Nutrition Centre, Hills Road, Cambridge CB2 2DH, UK.*

**Summary** Intakes of starch, non-starch polysaccharides (NSPs), protein and fat have been compared with colorectal cancer incidence in 12 populations worldwide. There were strong inverse associations between starch consumption and large bowel cancer incidence (large bowel $r = -0.70$, colon $r = -0.76$). There was no significant relation with NSPs, although the association with large bowel cancer incidence was still significant when NSP was combined with resistant starch (RS) to give an estimate of fermentable carbohydrate (large bowel $r = -0.52$, colon $r = -0.60$). The relationships between starch, RS and NSPs and cancer incidence remained statistically significant after adjusting for fat and protein intakes. The strong inverse associations found here suggest a potentially important role for starch in protection against colorectal cancer and correspond with the hypothesis that fermentation in the colon is the mechanism for preventing colorectal cancer. Measures of both starch and NSPs need to be included in future epidemiological studies of diet and bowel cancer.

In epidemiological studies of colorectal cancer, diet is strongly associated with colorectal incidence rates. Cross-sectional, case-control and prospective data demonstrate increased risks for high meat and fat consumption, and a reduction in risk for individuals and populations consuming high amounts of dietary fibre and vegetables (Bingham, 1990; Tomatis, 1990). The attributable population risk from low dietary fibre consumption is presently estimated to be 35% (Tomatis, 1990).

The recommended definition of dietary fibre is non-starch polysaccharides (NSPs) (Department of Health, 1991). NSPs escape digestion in the small bowel and are then largely fermented by bacteria in the colon with the production of short-chain fatty acids (acetate, propionate and butyrate). Bacterial growth is stimulated, which, together with any unfermented NSPs, leads to an increase in stool weight, dilution of colonic contents and faster transit time through the large gut (Stephen & Cummings, 1980). Recent studies have shown an inverse association between high stool weight and colorectal cancer incidence (Cummings *et al.*, 1992*a*). It is through fermentation that NSPs are thought to protect against bowel cancer.

However, recent studies in man have shown that a significant amount of starch also escapes digestion in the small gut, depending on the physical form of food eaten, the granule type, and how it is cooked and processed (Englyst & Cummings, 1985, 1986, 1987). This starch, called resistant starch (RS), is again largely fermented in the colon, and has laxative properties similar to NSPs (Cummings *et al.*, 1992*b*). Starch may be particularly important as a protective factor in colorectal cancer because both *in vivo* and *in vitro* studies have shown that fermentation of starch increases the amount of butyrate formed in relation to other fatty acids (Englyst *et al.*, 1987; Scheppach *et al.*, 1988). Butyrate was first suggested as a protective factor in colorectal cancer because it is a major product of fermentation in the colon and it is known to suppress cell proliferation (Cummings *et al.*, 1981). It also inhibits histone deacetylation, leading to arrested growth in the $G_1$ phase and alteration of chromatin accessibility to DNA repair enzymes (Kruh, 1982; Smith, 1986). Butyrate induces differentiation in colon carcinoma cell lines (Whitehead *et al.*, 1986), and in rodents luminal butyrate levels are inversely associated with colonic cell proliferation, and positively associated with histone acetylation (Boffa *et al.*, 1992). High-starch diets fed to mice have also been shown to reduce proliferative activity in the colon (Caderni *et al.*, 1989).

Correspondence: A. Cassidy.
Received 4 March 1993; and in revised form 14 December 1993.

Although starch intake is frequently disregarded in dietary surveys, it is possible that RS may be a major protective factor against colorectal cancer. In a correlational study we have therefore determined the epidemiological relationship between total starch intakes, RS and NSPs and large bowel cancer risks.

### Methods

There is considerable international variability in survival for large bowel cancer, thus incidence data were considered more appropriate than mortality data for this study. Published cancer incidence data are currently available for 31 countries worldwide (Muir *et al.*, 1987). Dietary information from population samples of adult men and women living in these countries was then obtained. The Indian dietary data were obtained in Bangalore, and Bangalore incidence data were used in the analysis. However, all other food intake data were obtained from national samples, and mean national cancer incidence rates were calculated from all registry data available for each country. All-age male and female colon and rectal cancer rates, age standardised to world populations, were used to calculate mean national cancer incidence rates for each country.

Information on starch, NSP, fat and protein intake had previously been gathered by the authors and their collaborators for UK, Denmark, Finland and Japan (IARC Large Bowel Cancer Group, 1983; Kuratsune *et al.*, 1986; Bingham *et al.*, 1990). To extend this information, a Medline search of all publications indexed since 1975 was made for references to dietary intakes of 'starch', 'carbohydrate', 'lipid', 'protein' and 'dietary fibre'. A hand search of the 'dietary studies' section in each issue of *Nutrition Abstracts and Reviews* from 1975 to the present was then carried out. The 'carbohydrate', 'lipid', 'protein' and 'food intake' sections were also examined. This left 25 counries for which no suitable data were forthcoming. A letter was therefore sent to a scientist known to the investigators in each of these counries asking for recent information on dietary intakes of NSPs, starch, fat and protein for adult men and women, preferably from randomly selected population samples. If this information was not available, detailed average food consumption data were requested to calculate NSP and starch intakes (Paul & Southgate, 1978; Englyst *et al.*, 1988, 1989). The amount of RS in the diet is more difficult to determine because analytical estimates of RS in specific foods published in food tables record only retrograded amylose (Holland *et al.*, 1988). In the limited number of foods that have been investigated by an *in vitro* technique 1-75% of dry matter is present as RS (Englyst *et al.*, 1992). Direct measures indicate that 4–10% of starch in mixed diets reaches the large gut (Stephen *et al.*, 1983; Flourie *et al.*, 1988). In order to obtain an estimate of RS and NSPs reaching the large gut, we have therefore taken a conservative estimate of 5% total starch as RS and combined this with NSPs. Estimates were restricted to those populations with large bowel cancer incidence data (Muir *et al.*, 1987) and, because of analytical differences between methods, only those analyses measuring dietary fibre as NSPs have been used.

Statistical analysis of dietary intakes and colorectal cancer incidence was performed using Systat 5.1; Pearson correlation coefficients and multiple regression were used.

### Results

Three reports found in the search of *Nutrition Abstracts and Reviews* provided sufficient dietary information for inclusion in this study (Calkins *et al.*, 1984; Kaufmann *et al.*, 1986; Junshi *et al.*, 1991). Of the remaining 25 countries contacted by letter, six did not reply and ten were at that time unable to supply any dietary information on NSP and starch intakes. Contacts in Australia (K. Baghurst, personal communication), Japan (S. Nakaji, personal communication) and The Netherlands (K. Hulshof, personal communication) provided information on average food consumption, and from these data mean intakes of NSPs and/or starch were calculated and intakes of fat and protein were provided. Dietary intakes of starch, fat and protein were provided for Norwegian adults by M. Nes (personal communication). The Irish National Nutrition Survey provided sufficient information for the calculation of NSP intakes, and supplied data on fat, protein and starch intakes (Lee & Cunningham, 1990), while the OPCS 'The Dietary and Nutritional Survey of British Adults' study (Gregory *et al.*, 1990) supplied data on daily intakes of fat, starch and protein in British adults. Dietary intakes of NSPs, starch, protein and fat were provided for Indian adult males and females by P. Shetty (personal communication).

Table I shows age-standardised (world) rates per 100,000 for colon, rectum and large bowel cancer in the 11 countries (28 groups) for which appropriate dietary information was obtained. Colon and large bowel cancer incidence was highest for Australian men and lowest for Indian females. The table also shows mean intakes of NSPs, starch, fat and protein for adult men and women in each of these countries. Calculated NSP intakes ranged from 10.9 g $day^{-1}$ to 21.0 g $day^{-1}$ for males and from 9.0 g $day^{-1}$ to 15.5 g $day^{-1}$ for females. Starch intake was lowest in Australia for men (102 g $day^{-1}$) and lowest in the USA for females (73 g $day^{-1}$) and highest in China (371 g $day^{-1}$). The coefficient of variation between countries in protein (15%) and fat (28%) intake was smaller than for starch (50%). The coefficient of variation in NSP intake between countries was 20%. Mean intakes of all variables were greater in men than women.

Pearson correlation coefficients between each dietary variable and incidence rates of colon, rectal or large bowel cancer are shown in Table II. Significant inverse associations ($r = -0.74$ to $-0.86$) between starch intake and cancer of the colon, rectum and large bowel were present for men, and for colon and large bowel in women. Figure 1 shows the association for the male and female data combined for starch and colon cancer ($r = -0.76$). Inverse associations were also shown for NSPs, but these were much weaker and not statistically significant. However, significant inverse associations were found when rates were related to total fermentable polysaccharides (NSPs + RS; $r = -0.70$ to $-0.93$), particularly for females. Data for the sexes combined related less well.

Positive associations between colon and large bowel cancer incidence and protein intake were observed for men but not for women (Table II). Fat consumption was weakly related to colon and large bowel cancer incidence in women, but only to large bowel cancer for men (Table II).

In multiple regression analysis (Table III), after adjusting for fat and protein intake, the significant relationships between cancer incidence and intake of starch and NSPs + RS remained. For example, when the male and female data were combined, starch intakes were still significantly related to colon ($t = 5.27$, $P < 0.001$) and large bowel cancer incidence ($t = 4.53$, $P < 0.001$) and, to a lesser extent, NSPs + RS intake was still significantly related to colon ($t = -4.27$, $P < 0.001$), rectal ($t = -3.46$, $P < 0.01$) and large

**Table I** Dietary intake (g $day^{-1}$) and cancer incidence (cases per 100,000 $year^{-1}$; age standardised, world) in various populations

| *Country* | *Sex* | *n* | *Method* | *Reference* | *Starch* | *NSP* | *Protein* | *Fat* | *Colon* | *Rectum* | *Large bowel* |
|---|---|---|---|---|---|---|---|---|---|---|---|
| Australia | M | 1,000 | FFQ | Baghurst (PC) | 101.9 | 13.2 | 89.2 | 90.2 | 24.9 | 16.6 | 40.4 |
| | F | 1,000 | FFQ | Baghurst (PC) | 81.2 | 12.0 | 76.4 | 73.0 | 22.0 | 10.2 | 32.5 |
| China[a] | M | | 3 d WI | Junshi *et al.* (1991) | 371.0 | — | 65.8 | 44.2 | 6.3 | 7.9 | 14.2 |
| | F | | 3 d WI | Junshi *et al.* (1991) | 371.0 | — | 65.8 | 44.2 | 6.0 | 6.8 | 12.8 |
| Denmark | M | 60 | 4 d WI/DD | IARC 1982 | 127.0 | 15.6 | 88.5 | 130.5 | 18.9 | 17.4 | 36.3 |
| Finland | M | 59 | 4 d WI/DD | IARC 1982 | 220.0 | 16.5 | 100.5 | 117.5 | 10.0 | 10.1 | 20.1 |
| India[b] | M | 16 | 7 d WI | Shetty (PC) | 309.2 | 21.0 | 66.8 | 83.4 | 2.2 | 3.4 | 5.6 |
| | F | 15 | 7 d WI | Shetty (PC) | 215.2 | 15.5 | 44.2 | 57.1 | 1.8 | 3.0 | 4.9 |
| Ireland | M | 305 | 7 d DH | Lee 1990 | 189.8 | 11.0 | 100.3 | 109.8 | 17.8 | 13.0 | 30.8 |
| | F | 371 | 7 d DH | Lee 1990 | 124.3 | 9.0 | 69.5 | 73.3 | 17.1 | 8.6 | 25.7 |
| Israel | M | 285 | 24 h recall | Kaufmann *et al.* (1986) | 143.7 | — | 79.1 | 82.4 | 14.6 | 15.5 | 30.0 |
| | F | 193 | 24 h recall | Kaufmann *et al.* (1986) | 88.7 | — | 56.8 | 62.3 | 14.6 | 12.2 | 26.8 |
| Japan | M | – | DD | Kuratsune *et al.* (1986) | | 10.9 | | | 12.9 | 11.2 | 21.3 |
| | F | – | DD | Kuratsune *et al.* (1986) | | 10.9 | | | 9.2 | 6.9 | 16.4 |
| | M | | NNS | Nakaji (PC) | 136.5 | — | 79.2 | 58.3 | | | |
| | F | | NNS | Nakaji (PC) | 136.5 | — | 79.2 | 58.3 | | | |
| Netherlands | M | 1930 | 2 d record | Hulshof (PC) | 143.3 | 15.9 | 86.5 | 118.3 | 20.6 | 15.0 | 35.6 |
| | F | 2204 | 2 d record | Hulshof (PC) | 99.4 | 13.1 | 69.5 | 93.0 | 19.7 | 9.1 | 28.7 |
| Norway | M | | HFS | Nes (PC) | 140.0 | — | 77.0 | 98.0 | 17.4 | 14.8 | 32.2 |
| | F | | HFS | Nes (PC) | 140.0 | — | 77.0 | 98.0 | 17.7 | 10.2 | 27.9 |
| UK | M | 32 | 7 d WI | Bingham *et al.* (1990) | | 11.2 | | | 18.6 | 13.5 | 32.0 |
| | F | 31 | 7 d WI | Bingham *et al.* (1990) | | 12.5 | | | 16.8 | 8.1 | 24.9 |
| | M | 1,087 | 7 d WI | Gregory *et al.* (1990) | 156.0 | — | 84.7 | 102.3 | | | |
| | F | 1,110 | 7 d WI | Gregory *et al.* (1990) | 106.0 | — | 62.0 | 73.5 | | | |
| USA | M | 1,013 | 24 h recall | Kaufmann *et al.* (1986) | 111.5 | | | | 27.9 | 14.2 | 42.0 |
| | F | 1,229 | 24 h recall | Kaufmann *et al.* (1986) | 72.9 | | | | 22.0 | 9.4 | 31.4 |
| | M | 25 | 3 d WI/DD | Calkins *et al.* (1984) | | 17.1 | 106.0 | 109.0 | | | |
| | F | 25 | 3 d WI/DD | Calkins *et al.* (1984) | | 12.1 | 62.0 | 70.0 | | | |

[a]Nationwide survey. [b]Indian dietary and incidence data from Bangalore. DD, duplicate diet; WI, weighed intakes; DH, diet history; NNS, national nutrition survey; HFS, household food survey; PC, personal communication.

bowel cancer incidence ($t = -4.79$, $P<0.001$). The relation with NSPs remained statistically insignificant.

Fat intake was weakly related or unrelated to colorectal cancer incidence (Table II). However, in multiple regression analysis when the effect of fat was allowed to be modified by the level of starch (statistical interaction) then fat became very important (Table III). There is no evidence from this study of a protein modifying effect. NSPs and NSPs + RS had no modifying effect on fat intake.

**Figure 1** The association between starch intake (g day$^{-1}$) and colon cancer incidence (males and females combined, $n = 22$) (cases per 100,000 age-standardised world population year$^{-1}$).

### Discussion

A number of previous cross-sectional studies have made international comparisons between dietary intake and colorectal cancer incidence (Drasar & Irving, 1973; Armstrong & Doll, 1975; McKeown-Eyssen & Brightsee, 1985; Brightsee & Jazmaji, 1991). These comparisons have all made use of food balance sheets, but these data do not measure food actually consumed by the population and make only limited corrections for wastage, which is likely to vary from one population to another. Moreover, differences in food inake between different age groups and sexes cannot be examined in relation to cancer incidence. These imperfect data generally all indicate strong positive associations between fat and animal protein consumption and bowel cancer incidence or mortality.

There are no international comparisons using food balance sheets of NSPs and bowel cancer incidence or mortality, although McKeown-Eyssen and Brightsee (1985) correlated dietary fibre intakes, calculated using older food table analyses, and population data from food balance sheets with

**Table II** Pearson correlation coefficients between dietary intake of starch, NSPs, protein and fat and incidence of colorectal cancer

| | *NSPs* | *Starch* | *NSPs + RS* | *Fat* | *Protein* |
|---|---|---|---|---|---|
| Colon cancer | | | | | |
| Males | − 0.36 (NS) ($n$ = 9) | − 0.84*** ($n$ = 12) | − 0.71* ($n$ = 9) | 0.51 (NS) ($n$ = 12) | 0.67* ($n$ = 12) |
| Females | − 0.44 (NS) ($n$ = 7) | − 0.76** ($n$ = 10) | − 0.85** ($n$ = 7) | 0.69* ($n$ = 10) | 0.47 (NS) ($n$ = 10) |
| Males and females | − 0.29 (NS) ($n$ = 16) | − 0.76*** ($n$ = 22) | − 0.60* ($n$ = 16) | 0.53** ($n$ = 22) | 0.51* ($n$ = 22) |
| Rectal cancer | | | | | |
| Males | − 0.48 (NS) ($n$ = 8) | − 0.86*** ($n$ = 12) | − 0.79** ($n$ = 9) | 0.51 (NS) ($n$ = 12) | 0.48 (NS) ($n$ = 12) |
| Females | − 0.59 (NS) ($n$ = 7) | − 0.56 (NS) ($n$ = 10) | − 0.93*** ($n$ = 7) | 0.46 (NS) ($n$ = 10) | 0.45 (NS) ($n$ = 10) |
| Males and females | − 0.11 (NS) ($n$ = 16) | − 0.47* ($n$ = 22) | − 0.31 (NS) ($n$ = 16) | 0.64** ($n$ = 22) | 0.66*** ($n$ = 22) |
| Large bowel cancer | | | | | |
| Males | − 0.37 (NS) ($n$ = 8) | − 0.86*** ($n$ = 12) | − 0.71* ($n$ = 9) | 0.56* ($n$ = 12) | 0.63* ($n$ = 12) |
| Females | − 0.49 (NS) ($n$ = 7) | − 0.74** ($n$ = 10) | − 0.88** ($n$ = 7) | 0.65* ($n$ = 10) | 0.49 (NS) ($n$ = 10) |
| Males and females | − 0.23 (NS) ($n$ = 16) | − 0.70*** ($n$ = 22) | − 0.52* ($n$ = 16) | 0.62** ($n$ = 22) | 0.60** ($n$ = 22) |

*$P<0.05$, **$P<0.01$, ***$P<0.001$. NS, not significant. RS estimated as 5% starch intake. $n$, number of data points.

**Table III** $t$-values of multiple regression analysis after adjusting for fat and protein intakes and after interaction term analysis

| | *Starch* | *NSPs* | *NSPs + RS* | *Fat (interaction with starch)* |
|---|---|---|---|---|
| Males | | | | |
| Colon cancer | − 3.31** | − 0.73 (NS) | − 2.07 (NS) | 2.80* |
| Rectal cancer | − 3.79** | − 1.74 (NS) | − 4.91** | 3.03* |
| Large bowel cancer | − 3.43** | − 1.03 (NS) | − 2.70* | 4.36** |
| Females | | | | |
| Colon cancer | − 2.04 (NS) | − 0.52 (NS) | − 2.73 (NS) | 3.43* |
| Rectal cancer | − 1.23 (NS) | − 0.64 (NS) | − 3.23* | 1.11 (NS) |
| Large bowel cancer | − 1.99 (NS) | − 0.69 (NS) | − 2.76 (NS) | 2.78* |
| Males and females | | | | |
| Colon cancer | − 5.27*** | − 1.79 (NS) | − 4.27*** | 3.75** |
| Rectal cancer | − 2.03 (NS) | − 2.04 (NS) | − 3.46** | 2.67* |
| Large bowel cancer | − 4.53*** | − 2.04 (NS) | − 4.79*** | 4.71*** |

*$P<0.05$, **$P<0.01$, ***$P<0.001$. NS, not significant.

colon cancer mortality rates in 38 countries. They reported higher estimates of dietary fibre intake in low colon cancer risk countries ($r = -0.36$). However, because of the strong ($r = 0.88$, 0.74) correlations with meat and fat, the fibre correlations were not significant when multiple regression analysis, adjusting for meat and fat intake, was performed ($r = -0.18$ and $-0.36$ respectively).

In the present study positive associations between fat, protein and large bowel cancer were also found, although correlations were generally smaller than those reported by McKeown-Eyssen and Brightsee (1985) ($r = 0.56-0.65$, Table II). NSP intakes were also inversely associated with bowel cancer, but the correlations were not significant (Table II) and weaker than those with fat and protein. When adjusted for fat and protein intake, the associations with NSP remained insignifiant (Table III).

However, we have previously shown, in a study of bowel cancer mortality within the UK using data from The National Food Survey, a survey of households, that even after controlling for fat, beef and protein intakes the protective associations with NSPs and vegetables are independently related to bowel cancer (Bingham et al., 1979, 1985). Significant inverse relations between bowel cancer and intakes of dietary fibre for NSPs were also obtained in two studies of geographical areas at differing risk of colorectal cancer within Scandinavian populations (IARC Large Bowel Cancer Group, 1982; Rosen et al., 1988). Case–control studies of individuals within populations also generally show a reduction in relative risk for individuals consuming more NSPs (Bingham, 1990; Tomatis, 1990). Within populations at high risk for bowel cancer, from high meat and fat consumption, reduced consumption of dietary fibre is therefore associated with increased risk (Tomatis, 1990). Willett et al. (1990) were unable to detect a protective association between dietary fibre and colorectal incidence in their prospective study of US nurses. However, in a later prospective study of adenomas, in which an extended dietary questionnaire was used, risk of individuals in the highest quintile of fibre intake relative to the lowest was 0.36 ($P < 0.001$) (Giovannucci et al., 1992).

Fibre is not the only possible protective item in food, and neither is it the only substrate for fermentation in the large bowel. Intakes of starch are usually 8–10 times higher than intakes of NSPs (Table I), and a significant proportion of starch reaches the large bowel. Consumption of starch in relation to large bowel cancer has not previously been considered in international comparisons. Brightsee and Jazmaji (1991) estimated starch availability from food balance sheets. Starch intakes varied internationally over a 3-fold range, from 139 g per day in Iceland to 386 g in Yugoslavia, but the authors did not relate these data to cancer risk. In general, the individual estimates reported here, in which wasted food is not included, also varied 3-fold but yielded lower values with an overall mean of 164 g day$^{-1}$, compared with an average of 223 g day$^{-1}$ found by Brightsee and Jazmaji (1991) in the same countries. This compares with an average of 14 g day$^{-1}$ NSPs estimated from the present data.

Starch intake data on only a few populations were gathered in this investigation. Information on starch intake is rarely reported in the literature, because most papers confine their reports to total carbohydrate estimates. Older food tables estimated carbohydrates 'by difference' and direct measurement of sugars and starches was not undertaken. The increasing recognition of the different physiological properties of starch and sugars in nutrition should prompt a greater variety of food analyses in which the different carbohydrates are separately identified.

There are known differences in faecal weight and transit time between sexes, and differences in colorectal cancer incidence by subsite which have been linked to differences in metabolism of the sex hormones (McMichael & Potter, 1983; Cummings et al., 1992a). Confounding of sex may therefore have accounted for the lower correlations for the combined estimates compared with the sex-specific estimates (Table II). Short-chain fatty acids are rapidly absorbed from the colon, with little difference in the molar ratios of acetate–propionate–butyrate in the distal versus the proximal colonic contents (Cummings et al., 1987). It is probable that starches fermented at a slow rate would be of particular benefit in cancer protection in the distal colon and rectum, where butyrate is specifically utilised by these populations.

Owing to the paucity of data in food tables, there are very few epidemiological studies reported which have assessed starch consumption in relation to colorectal cancer risk. Tuyns et al. (1987), in one of the largest case–control studies so far reported, found reductions in relative risk to 0.82 (not significant) and 0.67 for increased levels of polysaccharides and fibre consumption respectively. No significant trends for starch were observed in other case–control studies in Utah and Russia (Slattery et al., 1988; Zaridze et al., 1993).

The present study is the first to examine international associations between starch consumption and large bowel cancer. The associations with starch were strong (large bowel cancer $r = 0.70$; colon cancer $r = -0.76$). We have assumed as a conservative estimate that 5% of total starch would enter the large bowel, and when combined with NSPs to give RS and NSPs entering the colon, the association with cancer incidence was weaker but still significant, $r = -0.60$ for colon cancer and $r = 0.52$ for large bowel cancer. These relationships between polysaccharides and cancer incidence remained statistically significant after controlling for fat and protein intakes.

Omission of important confounding variables from the statistical analysis can lead to bias in estimating exposure effects if those covariates are associated with exposure and disease (Greenland, 1992). One important class of covariates are 'effect modifiers', therefore our statistical analysis included interaction terms. The results of this analysis suggest that starch intake modifies the effect of fat. Therefore, starch appears to protect against fat intake in relation to colorectal cancer. All of these results suggest that epidemiological and experimental investigations need to take account of starch, RS and NSP intakes in future investigations.

Although ecological correlation studies are generally considered as suggestive rather than investigative, in nutritional epidemiology 'ecological studies are ideal for examining new, *a priori* hypotheses and may lead on to studies of individuals from which causality may be inferred with greater confidence' (Margetts, 1991). One of the main limitations of the current study is the problem of matching the cancer incidence data with appropriate dietary information. In as far as was possible, the cancer incidence data used in the analysis covered the same population from which the diet data were obtained. The cancer incidence data in China rely on data from three urban regions, however even when the China data were removed the correlation coefficients observed were similar (for example, colon cancer and starch: $r = -0.86$ males, $r = -0.88$ females and $r = -0.75$ males and females combined).

The strong inverse associations found here, together with other data, suggest an important, though not exclusive, role for starch in protection against colorectal cancer. Our hypothesis is that fermentation in the colon is the mechanism for achieving colorectal cancer protection, via the specific contribution of butyrate to reduction of proliferation and induction of differentiation (Cummings et al., 1981). This, together with more recent studies demonstrating a preference for butyrate production by the bacterial flora when starch is the main substrate for fermentation (Englyst et al., 1987), the documented laxative effects of starch (Cummings et al., 1992b) in addition to those of NSPs (Cummings et al., 1992a) and the protective relation between increased stool weight and colorectal cancer (Cummings et al., 1992a) all suggest a mechanism for the epidemiological associations.

The authors gratefully acknowledge information on starch, NSPs, and food consumption provided by Dr K. Baghurst (Australia), Dr S. Nakaji (Japan), Professor P. Shetty (India) and K. Hulshof (R D

The Netherlands). Dr T. Cole is thanked for statistical assistance. All of the scientists (Dr M. Nes, Norway; Dr A. Stephen and Dr R. Gibson, Canada; Dr J. Hankin, Hawaii; Dr L. Kohlmeier, Dr H. Boeing and G. Winkler, Germany; Dr M. Minowa, Japan; Dr W. Becker, Sweden; Dr L. Steingrimsdottir, Iceland; and Dr M. De Guzman, Philippines) who forwarded dietary information are also thanked. Dr G. Neale, Department of Gastroenterology, is thanked for financial assistance.

### References

ARMSTRONG, B. & DOLL, R. (1975). Environmental factors and cancer incidence in different countries with special references to dietary practices. *Int. J. Cancer*, **15**, 617–631.

BINGHAM, S. (1990). Mechanisms and experimental evidence relating dietary fibre (NSP) and starch to protection against large bowl cancer. *Proc. Nutr. Soc.*, **49**, 153–171.

BINGHAM, S., WILLIAMS, D.R.R., COLE, T.J. & JAMES, W.P.T.J. (1979). Dietary fibre and regional large bowel cancer mortality in Britain. *B. J. Cancer*, **40**, 456–463.

BINGHAM, S., WILLIAMS, D.R.R. & CUMMINGS, J.H. (1985). Dietary fibre consumption in Britain: new estimates and their relation to large bowel cancer mortality. *B. J. Cancer*, **52**, 399–402.

BINGHAM, S.A., PETT, S. & DAY, K. (1990). Non-starch polysaccharide intake of a representative sample of British adults. *J. Hum. Nutr. Dietet.*, **3**, 333–337.

BOFFA, L.C., LUPTON, J.R., MARIANI, M.R., CEPPI, M., NEWMARK, H., SCALMATI, A. & LIPKIN, M. (1992). Modulation of colonic cell proliferation, histone acetylation and luminal short chain fatty acids by variation of dietary fiber (wheat bran) in rats. *Cancer Res.*, **52**, 5906–5912.

BRIGHTSEE, E. & JAZMAJI, V. (1991). Estimation of the amount of dietary starch available to different populations. *Can. J. Physiol. Pharmacol.*, **69**, 56–59.

CADERNI, G., BIANCHINI, F., DOLORA, P. & KREIBEL, D. (1989). Proliferative activity in the colon of the mouse and its modulation by dietary starch, fat, and cellulose. *Cancer Res.*, **49**, 1655–1659.

CALKINS, B.M., WHITTAKER, D.J., NAIR, P.P., RIDER, A.A. & TURJMAN, N. (1984). Diet, nutrition intake and metabolism in populations at high and low risk for colon cancer. *Am. J. Clin. Nutr.*, **40**, 896–905.

CUMMINGS, J.H., STEPHEN, A.M. & BRANCH, W.J. (1981). Implications of dietary fiber breakdown in the human colon. In *Banbury Report 7 Gastrointestinal Cancer*, Bruce, W.R., Correa, P., Lipkin, M., Tannenbaum, S. & Wilkins, T.D. (eds), pp. 71–81. Cold Spring Harbor Laboratory Press: Cold Spring Harbor, NY.

CUMMINGS, J.H., POMARE, E.W., BRANCH, W.J., NAYLOR, C.P.E. & MACFARLANE, G.T. (1987). Short chain fatty acids in human large intestine, portal, hepatic and venous blood. *Gut*, **28**, 1221–1227.

CUMMINGS, J.H., BINGHAM, S.A., HEATON, K.W. & EASTWOOD, M.A. (1992*a*). Fecal weight, colon cancer and dietary intake of NSP (dietary fibre). *Gastroenterology*, **103**, 1783–1789.

CUMMINGS, J.H., BEATTY, E.R., KINGMAN, S., BINGHAM, S. & ENGLYST, H.N. (1992*b*). Laxative properties of resistant starches. *Gastroenterology*, **102**, A548.

DEPARTMENT OF HEALTH (1991). Dietary reference values for food, energy and nutrients for the UK. In *Report on Health and Social Subjects*, Vol. 41. HMSO: London.

DRASAR, B.S. & IRVING, D. (1973). Environmental factors and cancer of the colon and breast. *Br. J. Cancer*, **27**, 167–172.

ENGLYST, H.N. & CUMMINGS, J.H. (1985). Digestion of the polysaccharides of some cereal foods in the human small intestine. *Am. J. Clin. Nutr.*, **42**, 778–787.

ENGLYST, H.N. & CUMMINGS, J.H. (1986). Digestion of the carbohydrates of banana in the human small intestine. *Am. J. Clin. Nutr.*, **44**, 42–50.

ENGLYST, H.N. & CUMMINGS, J.H. (1987). Digestion of the polysaccharides of potato in the human small intestine. *Am. J. Clin. Nutr.*, **45**, 423–431.

ENGLYST, H.N., HAY, S. & MACFARLANE, G.T. (1987). Polysaccharide breakdown by mixed populations of human faecal bacteria. *FEMS Microbiol. Ecol.*, **95**, 163–171.

ENGLYST, H.N., BINGHAM, S.A., RUNSWICK, S.A., COLLINSON, E. & CUMMINGS, J.H.C. (1988). Dietary fibre (non-starch polysaccharides) in fruit, vegetables and nuts. *J. Hum. Nutr. Dietet.*, **2**, 253–271.

ENGLYST, H.N., BINGHAM, S.A., RUNSWICK, S.A., COLLINSON, E., CUMMINGS, J.H.C. (1989). Dietary fibre (non-starch polysaccharides) in cereal products. *J. Hum. Nutr. Dietet.*, **1**, 247–286.

ENGLYST, H.N., KINGMAN, S.M. & CUMMINGS, J.H. (1992). Classification and measurement of nutritionally important starch fractions. *Eur. J. Clin. Nutr.*, **46**, S33–S50.

FLOURIE, B., LEBLOND, A., FLORENT, C., RAUTURAEU, M., BISALLI, A. & RAMBAUD, J.C. (1988). Starch malabsorption and breath gas excretion in healthy humans consuming low and high starch diets. *Gastroenterology*, **95**, 356–363.

GIOVANNUCCI, E., STAMPFER, M.J., COLDITZ, G., RIMM, E.G. & WILLETT, W.C. (1992). Relation of diet to the risk of colorectal adenoma in men. *J. Natl Cancer Inst.*, **84**, 91–98.

GREENLAND, S. (1992). Divergent biases in ecologic and individual level studies. *Stats Med.*, **11**, 1209–1223.

GREGORY, J. FOSTER, K., TYLER, H. & WISEMAN, M. (1990). *The Dietary and Nutritional Survey of British Adults.* Office of Population Censuses and Surveys. HMSO: London.

HAENZEL, W., BERG, J.W., SGI, M., KURIHARA, M. & LOCK, F.B. (1973). Bowel cancer in Hawaii Japanese. *J. Natl Cancer Inst.*, **51**, 1765–1779.

HOLLAND, B., UNWIN, I.D. & BUSS, D.H. (1988). Cereals and cereal products. Third supplement to *The Composition of Foods*, 4th edn. Royal Society of Chemistry: Letchworth, Herts.

IARC LARGE BOWEL CANCER GROUP (1982). Second IARC international collaborative study on diet and large bowel cancer in Denmark and Finland. *Nutr. Cancer*, **4**, 3–79.

JUNSHI, C., CAMPBELL, T.C., JUNYAOL, L. & PETO, R. (eds) (1991). *Diet Lifestyle and Mortality in China. A Study of the Characteristics of 65 Chinese Counties.* Oxford University Press: Oxford.

KAUFMANN, N.A., DENNIS, B.H., HEISS, G., FRIEDLANDER, Y., KARK, J.D. & STEIN, Y. (1986). Comparison of nutrient intake of selected populations in the United States and Israel. Lipid Research Clinics prevalence study. *Am. J. Clin. Nutr.*, **43**, 604–619.

KRUH, J. (1982). Effect of sodium butyrate, a new pharmacological agent, on cells in culture. *Mol. Cell. Biochem.*, **42**, 65–82.

KURATSUNE, M., HONDA, T., ENGLYST, H.N. & CUMMINGS, J.H.C. (1986). Dietary fiber in the Japanese diet as investigated in connection with colon cancer risk. *Jpn J. Cancer Res.*, **77**, 736–738.

LEE, P. & CUNNINGHAM, K. (1990). *Irish National Food Survey.* Dublin: Irish Nutrition and Dietetic Institute.

MARGETTS, B.M. (1991). Basic issues in interpreting epidemiological research. In *Design Concepts in Nutritional Epidemiology*, Margetts, B.M. & Nelson, M. (eds), pp. 18–19. Oxford.

MCKEOWN EYSSEN, G. & BRIGHTSEE, E. (1985). Dietary factors in colon cancer: international relationships. An update. *Nutr. Cancer*, **7**, 251–253.

MCMICHAEL, A.J. & POTTER, J.D. (1983). Do intrinsic sex differences in lower alimentary tract physiology influence the sex-specific risks of bowel cancer and other biliary and intestinal diseases? *Am. J. Epidemiol.*, **118**, 620–627.

MUIR, C., WATERHOUSE, J., MACK, T., POWELL, J. & WHELAN, S. (eds) (1987). *Cancer Incidence in Five Continents*, Vol. V. IARC: Lyon.

PAUL, A.A. & SOUTHGATE, D.A.T. (1978). McCance and Widdowson's *The Composition of Foods*, 4th edn. HMSO: London.

ROEDIGER, W.E.W. (1982). Utilization of nutrients by isolated epithelial cells of the rat colon. *Gastroenterology*, **83**, 424–429.

ROSEN, M., NYSTROM, L. & WALL, S. (1988). Diet and cancer mortality in the counties of Sweden. *Am. J. Epidemiol.*, **127**, 42–49.

SCHEPPACH, W., FABIAN, C., SACHS, M. & KASPER, H. (1988). Effect of starch malabsorption on fecal SCFA excretion in man. *Scand. J. Gastroenterol.*, **23**, 755–759.

SLATTERY, M.C., SORENSON, A.W., MAHONEY, A.W., FRENCH, T.K., KRITCHEVSKY, D. & STREET, J.C. (1988). Diet and colon cancer: assessment of risk by fiber type and food source. *J. Natl Cancer Inst.*, **80**, 1474–1480.

SMITH, P.J. (1986). n-Butyrate alters chromatin accessibility to DNA repair enzymes. *Carcinogenesis*, **7**, 423–429.

STEPHEN, A.M. & CUMMINGS, J.H. (1980). Mechanisms of action of dietary fibre in the human colon. *Nature*, **284**, 283–284.

STEPHEN, A.M., HADDAD, A.C. & PHILLIPS, S.F. (1983). Passage of carbohydrate into the colon. Direct measurements in human. *Gastroenterology*, **85**, 589–595.

TOMATIS, L. (1990). *Cancer, Causes, Occurrence and Control.* IARC Scientific Publication No. 100, IARC: Lyon.

TUYNS, A.F., KAAKS, R. & HAELTERMAN, M. (1987). Colorectal cancer and the consumption of nutrients: a case-control study in Belgium. *Nutr. Cancer*, **10**, 181–196.

WHITEHEAD, R.H., YOUNG, G.P. & BHATHAL, B.S. (1986). Effects of short chain fatty acids on a new human colon carcinoma cell line (LIM1215). *Gut*, **27**, 1457–1463.

WILLETT, W.C., STAMPFER, M.J., COLDITZ, G.A., ROSNER, B.A. & SPIEZER, F.E. (1990). Relation of meat, fat and fibre intake to risk of colon cancer in a prospective study among women. *N. Engl. J. Med.*, **323**, 1664–1672.

ZARIDZE, D., FILIPCHENCKO, V., KUSTOV, V., SERDYUK, V. & DUFFY, S. (1993). Diet and colorectal cancer: results of two case-control studies in Russia. *Eur. J. Cancer*, **29A**, 112–115.